# From Committee on Medical Societies

**Published:** 1896-09

**Authors:** 


August 16, 1896.

*To the Physicians of Texas:*

At the last meeting of the State Medical Association, held in Fort Worth, a resolution was passed totally abrogating the initiation fees in joining that Association, five dollars for annual dues being the only expense now incurred. Presentation of diploma and compulsory attendance at the meetings are no longer required, the only prerequisite for membership is that the physician be in good standing with his local society, which local society must be in affiliation with the State Association.

Local medical societies must be regarded as branches of the parent stem. They should be united, as their objects are identical, and central action alone can enhance the value of the medical profession to humanity.

All local societies are earnestly solicited to become auxiliary to or affiliated with the State Association. Societies now in affiliation will please report to the secretary, Dr. H. A. West, Galveston, Texas, giving the names of officers and dates of meetings. This information should be sent at once, as it is important to publish it in the forthcoming Transactions, which will appear in about five weeks.

All members of local societies are urged to become members of the State Association, and lend their aid in upbuilding the same and perpetuating the medical work it has undertaken.

In territory where no local societies exist, any member of the State Association who may reside in such county or territory is requested to give a complete list of the physicians in his county or territory to the nearest society, so that the appeal may be made to them through the society.

Blank applications for membership will be furnished by the secretary, Dr. H. A. West, Galveston, Texas.

W. R. Blailock, McGregor, Chairman.
E. A. Woldert, Tyler.
L. L. Shropshire, San Antonio.
Joe D. Becton, McKinney.
S. E. Hudson, Austin.
Committee on Medical Societies.

